# Disability inclusion in higher education in Uganda: Status and strategies

**DOI:** 10.4102/ajod.v5i1.193

**Published:** 2016-12-02

**Authors:** Paul Emong, Lawrence Eron

**Affiliations:** 1 Department of Community and Disability Studies, Kyambogo University, Uganda; 2 Department of Special Needs Studies, Kyambogo University, Uganda

## Abstract

**Background:**

Uganda has embraced inclusive education and evidently committed itself to bringing about disability inclusion at every level of education. Both legal and non-legal frameworks have been adopted and arguably are in line with the intent of the Convention on the Rights of Persons with Disabilities (CRPD) on education. The CRPD, in Article 24, requires states to attain a right to education for persons with disabilities without discrimination and on the basis of equal opportunities at all levels of education.

**Objectives:**

Despite Uganda’s robust disability legal and policy framework on education, there is evidence of exclusion and discrimination of students with disabilities in the higher education institutions. The main objective of this article is to explore the status of disability inclusion in higher education and strategies for its realisation, using evidence from Emong’s study, workshop proceedings where the authors facilitated and additional individual interviews with four students with disabilities by the authors.

**Results:**

The results show that there are discrimination and exclusion tendencies in matters related to admissions, access to lectures, assessment and examinations, access to library services, halls of residence and other disability support services.

**Conclusion:**

The article recommends that institutional policies and guidelines on support services for students with disabilities and special needs in higher education be developed, data on students with disabilities collected to help planning, collaboration between Disabled Peoples Organisations (DPO’s) strengthened to ensure disability inclusion and the establishment of disability support centres.

## Introduction^1^

Uganda has embraced inclusive education and evidently committed itself to bringing about disability inclusion at every level of education. The commitment is demonstrated by the legal and non-legal frameworks on education and the establishment of educational infrastructure aimed at mainstreaming disability. The infrastructures include a department of special needs education at the Ministry of Education, Science, Technology and Sports, a special needs education section at the Uganda National Examinations Board, a department at the National Curriculum Development Centre, a section at the Education Standards Agency, representation of persons with disabilities at the National Council for Higher Education Board, Public Universities Councils and training of teachers for special needs education. The bulk of these infrastructures are visible in promoting inclusive education at primary and secondary levels of education.

The impact of the above developments is the increasing enrolment of students with disabilities in higher education being experienced recently. However similar infrastructures are not evident in higher education. There is however, affirmative action on admission of students with disabilities and other marginalized groups to public universities. Although this affirmative action is seen to be widening opportunities for students with disabilities to higher education, the law providing for it appears not to compel private universities^2^ to comply.

The right to education for students with disabilities in Uganda is still suffering from discrimination. Disability rights are often honoured in the breach (Lang, et al 2011), which leads to failure to achieve equal opportunities particularly in higher education.

This article examines the status of disability inclusion in higher education and strategies for its realisation in Uganda. Specifically, it explores experiences about disability inclusion in higher education, pointing out how discrimination and exclusion is demonstrated in admission, support services provided, access to libraries and halls of residence, lecture rooms, mode of delivery and mode of assessment.

### The right to education

The UN human rights law framework recognises education as a universal right and as enabling right to the attainment of other rights^3^. Denying an individual a right to education is arguably condemning such an individual to a denial or limitation in the enjoyment of fundamental rights. In general terms, the UN human rights law framework outlaws discrimination in education at all levels^4^ and comprehensively requires States to make educational services available, accessible, acceptable and adaptable,^5^ including to set minimum standards and to improve quality.^6^ These standards apply to people with disabilities as well by the principle of equality and non-discrimination, the cornerstone of the human rights law,^7^ based on the philosophy of inherent dignity and of the equal and inalienable rights of all human beings (Lauren, 2003).^8^ In examining the status and strategies for disability inclusion in higher education in Uganda, this article uses the foundation principles of inclusive education of equality, access and equal participation for all in every level of education (*source*). The critical question is what does each of these principles mean in regards to disability inclusion in education. This article provides an exploration of that using the social model of disability, the notion of non-discrimination and the intent of article 24 of the Convention on the Rights of People with Disabilities (CRPD) as benchmarks informing inclusive education in regards to disability.

A social model of disability is a theoretical understanding of the concept of disablement from a socio-political perspective (Oliver 2009:57). The argument is that disability is something imposed on people with disabilities on top of their impairment by an oppressive and discriminating social and institutional structure (UPIAS 1976:3–4). The *social model of disability mostly explains the relationship between people with impairments and their participation in society* (Oliver 1990:22). The model is premised on the principles that impairment and disability are distinctively different (UPIAS 1976 and Oliver 1996:4–5). The argument is that disability is a social oppression, not impairment, and that disability is a social construction, and to a large extent is culturally produced and culturally structured (Oliver 1996:22). For equal participation for people with disabilities, the model demands for the removal of the society’s economic, environmental, cultural and other barriers against people with disabilities (Barnes and Mercer 2010:30). The understanding of the social model of disability, in this article, brings about the operationalisation of the right to education for persons with disabilities without discrimination and on the basis of equal opportunities as enshrined in Article 24 of the CRPD.

The aim of Article 24 of the CRPD is to bring about an inclusive education system at all levels of education, with emphasis on understating the relationship between the learning environment and the impairment needs of a person with disability and the notion of non-discrimination. Based on Article 2 paragraph 3 of the CRPD the meaning of discrimination is wide enough to prohibit both intentional (direct) and non-intentional (indirect) discrimination or exclusion experienced by people with disabilities in society, including in education. Direct discrimination is discrimination which is intentional or overtly directed to particular individuals or groups. Direct discrimination is grounded on prejudices or stereotypes labeled on those group(s) of individuals. Indirect discrimination concerns non-intentional discrimination arising from practices which are neutral in nature but discriminatory in effect. Usually, these practices are embedded in institutional policies, norms and standards. In some jurisdictions, the concept indirect discrimination has since been developed to provide a broad scope of protection based on provision, criterion or practice (Monaghan, 2007:338). Arguably, the concepts ‘provisions, criterion or practice’ provide wide interpretation in relation to how higher education provides all arrangements for the students with disabilities. Indirect discrimination acknowledges the fact that problems of inequality are both systemic and simply individual in nature and therefore provides a picture of how groups are affected. The development of protection under indirect discrimination is arguably the major milestone towards achieving substantive equality (Meenan, 2007). In the perspective of disability, providing reasonable accommodation is one of the fundamental requirements for achieving substantive equality and is now a legal requirement of the CRPD. Reasonable accommodation means an essential practice to alleviate the disadvantage that arises for people with disabilities in the application of conventional requirements or systems (Schiek and Bell, 2007). The aim of reasonable accommodation is to bring about adaptation and change of the environment in order to remedy the detriment associated with the interaction between environment and impairment. In this article, the potential impact of reasonable accommodation is for institutions to adopt a proactive approach of avoiding discrimination against students with disabilities. Arguably, reasonable accommodation requires dismantling of systemic barriers in educational institutions arising from accessibility related challenges, ignorance of staff about specific disability needs, provisions and practices which are historically embedded in educational exclusion. It requires matching the needs to the appropriate support that brings about equal participation of students with disabilities in learning, participation and development.

The Ugandan legal frameworks^9^ are largely in line with the requirements of attaining the right to education provided by the United Nations Human Rights framework and fundamental principles of inclusive education. The Constitution of the Republic of Uganda (1995), Art 30 guarantees that all persons have a right to education. The intent of the constitution on higher education is reflected in the *Universities and Other Tertiary Institutions Act* (2001) (as amended) and the *Persons with Disability Act* (2006).

The *Universities and Other Tertiary Institutions Act* (2001) establishes the National Council for Higher Education (NCHE) of Uganda and details its mandates. The Act confers upon NCHE the responsibilities / functions of monitoring, evaluating, regulating and guiding the establishment of institutions of higher learning.^10^ The function of guiding obligates NCHE with the responsibility to ensure disability inclusion in institutions of higher learning. It also requires that NCHE certify that an institution of higher education has adequate and accessible physical structures.^11^ This function mandates NCHE to ascertain the extent to which physical accessibility of the institution’s facilities is ensured, in regards to disability^12^. Indeed, the Act empowers the NCHE to revoke a provisional license to an institution if it finds it not meeting the minimum requirements pertaining to physical infrastructure^13^. Unfortunately, in the Act, section 110- *Revocation of a Charter*, there is no mention about universal design and accessible facilities among the set grounds for such revocation. Failure by an institution to provide universal design, accessible facilities, reasonable accommodation, appropriate instruction or teaching methods and qualified staff for special needs students is a path to the exclusion of students with disabilities in the institution’s programmes.

On the composition of National Council for Higher Education (NCHE), the Act in section 7(1) (i) provides for representation on the Council by, among others, a person with disability appointed by, the Minister. Similarly, on composition of a university council of a public university, the Act provides that such a Council must be comprised of, among others, 2 representatives of persons with disabilities, one elected by members of staff and another by National Organisations of persons with disabilities.^14^ Definitely, such representation is aimed at creating awareness about disability inclusion to NCHE so that, in its regulatory role, NCHE ensures disability mainstream. On admission to public universities, section 28 of the Act provides for affirmative action for marginalized groups, including persons with disabilities. This is evidence that the Act gives the opportunity of acquiring higher education to all people wishing to do so, including persons with disabilities.^15^ In addition, the Act requires institutions to provide accessible physical facilities to the users of the public university.^16^ These are very noble objectives that expressly recognise persons with disabilities as among those who may wish to acquire higher education. However, the language in the Act is specific to public universities. This implies that the Act does not confer these obligations on private universities and other categories of institutions of higher learning. It is important to note, though, that some private universities admit students with disabilities based on their personal good will.

The *Persons with Disability Act* (2006), Part II guarantees a right to quality education to all learners with disabilities and special needs. It does this by conferring an obligation on government to promote educational development of persons with disabilities^17^ and prohibits their discrimination by all categories of educational institutions^18^. The Act imposes duties on bodies including institutions of higher learning to eliminate barriers to accessibility^19^ and prohibits discrimination in the provisions of goods, services and facilities of which higher education is a provider.^20^ The Act aims to develop an educational infrastructure that would guarantee an inclusive educational environment for all categories of people with disabilities^21^ through, among others, training of special needs teachers or personnel, formulation of and designing educational policies and programmes on inclusive education, providing structural and other adaptations in all educational institutions appropriate for the needs of persons with disabilities, committing not less than 10% of all educational expenditure to the educational needs of persons with disabilities, providing assistive devices suitable for students with special needs during examinations, including giving extra time. The Act explains discrimination against persons with disabilities in education as refusal or failure to accept an application for admission in an educational institution by a qualified person because of that person’s disability; or setting terms or conditions that exclude persons with disabilities; or by denying or limiting access to any benefits or service provided by the educational institution to a student with a disability; or expelling a student because of his or her disability; or by subjecting a student with disability to any other unfair treatment relating to his or her disability. The meaning of discrimination provided for by the Act prohibits either intentional or non-intentional discrimination as earlier discussed. However, the Act’s meaning of discrimination is short of requiring institutions to provide reasonable accommodation.

## The state of disability in higher education

Ugandan higher education has undergone reforms accruing from the structural adjustments economic policies experienced around the mid-1980s. The reforms saw the liberalization and privatization of the economy, including education, in the 1990s. The detailed discussions about these reforms and their effects on education are outside the scope of this article. However, suffice to state here that these reforms were aimed at fulfilling the critical need to meet the growing demand for higher education. The number of applicants at that time was estimated to be three times more than the available places (Kasozi, 2003) and there was need to reform the higher education sector to be relevant to the development needs of Uganda (Kasozi, 2005). These reforms have brought significant changes to higher education (Mamdani, 2007, Musisi & Muwanga, 2003, Kasozi, 2003). Quantitatively, there has been a rapid expansion of institutions of higher learning within two decades from less than 34 institutions^22^ to 164 institutions (32 universities and 132 tertiary institutions of education) by 2012 (UBOS, 2012) and increased number of students joining higher education (Bloom, Canning and Chan, 2006), including students with disabilities.

At the time of the reform, government had insufficient resources to provide for both basic and higher education, yet higher education in Uganda was largely financed and managed by the state. The government of Uganda then prioritised providing basic education and reduced its funding to higher education as a response to the global call for every State to ensure that every child’s right to basic education is met (UN, 1993). This largely contributed towards achieving an inclusive education at primary and secondary levels of education. The training of teachers in special needs education at Kyambogo University, a special educational needs unit at the Uganda National Examinations Board (UNEB), National Curriculum Development Centre (NCDC) and the inclusion of Special Needs Education component in the Primary Teachers’ College (PTC) curriculum were infrastructures put in place to ensure inclusive education. In higher education, similar infrastructures are lacking. Moreover, as a result of improved education environment for learners with disabilities at primary and secondary levels of education, over 1000 students with disabilities are joining higher education annually^23^.

Although higher education opened its doors for students with disabilities, little was done to incorporate the aspect of disability inclusion and reasonable accommodation at the initial stages of the reforms. Other than admitting students with disabilities through affirmative action by public universities, there is limited evidence of applying equal opportunities measures in other institutions of higher learning. These actions contravene the CRPD requirement which obliges states to ensure that institutions of higher learning adopt reasonable accommodation for persons with disabilities in all matters and arrangements an institution makes. For students with disabilities, reasonable accommodation implies arrangements necessary for their admissions, teaching, learning and assessment, library, accommodation, disability support provision, participation in sports and recreation. According to Emong (2014) it appears that the overall higher education environment is not changing in response to access requirements for admitted students with disabilities. He argues that institutions of higher learning lack disability policies, provide limited opportunities for admissions of candidates with disabilities, lack support services for students with disabilities and the libraries, accommodation, lectures, mode of delivery and mode of assessment are not easily accessible.

## Methodology

### Design and setting

This research was undertaken over a period of six months. The overall methodological design is descriptive qualitative study. The focus of the design is on the scope, implementation and impact of disability legislations in higher education in Uganda. The emphasis of this exploratory study is to gain insights (Denzin & Lincoln, 2000; Patton, 2002) and document voices and subjective human experiences (Silverman, 2010) of Uganda disability law in the text, as well as on various ideological and policy factors in respect of disability inclusion in higher education.

The study involved two levels. The first level was desk review of a study undertaken by Paul Emong on a similar topic in four Universities (2 governments and 2 private) in Uganda, analysis of education policies with a focus on higher education and statistical data on students with disabilities and other special needs at all levels. The second level gathered experiences on disability inclusion in higher education generated from two national workshops where the authors were facilitators and position papers drafted for Ugandan National Council for Higher Education, Vice Chancellors Forum and the Ministry of Education, Science, Technology and Sports of Uganda.

### Participants and procedure

Participants were drawn from among University staff and students with disabilities in Emong’s study. Altogether 46 University staff members from both science/medicine and humanities related faculties (*N* = 35 academic and 11 administrative staff) participated. A total of 121 students with disabilities were involved in the study. There were 5 focus group discussions of 10 student participants each, 14 student participants involved in in-depth interviews and 57 student participants filled questionnaires. The students with disabilities were drawn from the common disabilities in Uganda of physical disability, hearing impairment, visual impairment and other health related problems.

The participants in the national workshops were leaders of the National Disabled Peoples Organisations (*N* = 10), members of University top management (*N* = 3), representatives from National Council for Higher Education (*N* = 1), National Council for Disability (*N* = 1), Ministry of Gender, Labour and Social Development - where the docket of disability lies (*N* = 1) - and students with disabilities (*N* = 10).

A mixture of stratified, purposive and simple random sampling was used to draw participants in Emong’s study. The universities were purposively selected, drawing from both public and private universities known to have students with disabilities. A stratified sample of one University, Kyambogo University, was because of its specialty in disability, special needs education and rehabilitation training. The basis for purposively selecting University staff was their experience in working with students with disabilities. The teaching staff were at the rank of senior lecturers, heads of departments and deans of faculties. The administrative staff were particularly hall wardens who have direct interaction with students with disabilities. Sampled students were drawn from the five disability groups mentioned earlier. A simple random sample was thereafter used to select students with disabilities that filled in the questionnaire and those who participated in the focus group discussions. Purposive sampling of other students with disabilities helped to identify those who participated in in-depth interviews, picking from each category mild to severe disabling conditions. The national workshop participants were purposively selected based on their roles in the organisations and what they expressed about exclusion and discrimination of students with disabilities in higher education.

### Instruments

Four types of tools were used for data collection, namely focus group discussions, survey questionnaire, in-depth interview and workshop (presentation and feedback). They were designed to capture information based on key themes related to admissions, provision of support services, access to library services, access to lectures, mode of delivery and assessment, participation in sports and recreation, and physical environment accessibility by students with disabilities in Universities. A total of 117 questionnaires were sent out to students with disabilities in the four universities in Emong’s study; 57 were filled and returned. In-depth interviews were held with 14 students with disabilities and 46 university staff in Emong’s study. Another in-depth interview was held with 4 persons with disabilities identified during the workshop in the second level of the study. In Emong’s study 5 focus group discussions of 10 participants with disabilities each were held. Each focus group was composed of students with the same disability. A workshop approach was used by the authors to attain information as mentioned earlier from the other participants in level two of the study. Presentations of papers relating to disability inclusion in higher education were followed by discussions and recommended actions needed to bring about disability inclusion in higher education in Uganda.

### Data analysis

Information obtained from each of the instruments was analysed based on the themes. Closed ended and open ended questions in the questionnaire were coded and analysed using statistical Package for Social Science (SPSS) to generate descriptive data in Emong’s study. The information generated from the open ended questions was grouped based on the key issues it represents. The key issues were clustered according to themes as presented in the present article. Information obtained from focus group discussions, in-depth interviews and the workshop proceedings were recorded, transcribed into text, grouped into issues and themes generated. For purposes of strongly expressing issues on disability inclusion voices of participants are recorded.

## Ethical and Validity considerations

Ethical issues are present in all kind of research and arise at any stage. Effort was made to seek informed consent, assurance of confidentiality and privacy (Cottell & Dowine, 2000). While names of institutions and organisations are included in the study, there has been anonymity of the individual participants involved in the study. Validity does not belong to a separate stage in an investigation but permeates the entire research process (Kvale & Brinkmann, 2009). The process of control and rigour (Lincoln & Guba, 1985) was established by employing strict selection criteria, adequate sample size based on the population and data was double checked and returned to over and over again to see if the constructs, categories, explanations and interpretations make senses as presented in the excerpts of individual statements.

## Results and discussions

Four themes were identified to inform this article. The themes document experiences of the opportunities and challenges in admissions, support services, access to library and access to lectures, mode of delivery and assessment.

### Admission to higher education

Data indicated that students with disabilities were increasingly being admitted into institutions of higher education through different admission avenues. Students with disabilities and other special needs can access higher education on merit through the advanced level (high school) results commonly referred to as direct entry, the mature age entry scheme, the diploma/certificate scheme and the 64 slots government provides for admission of persons with disabilities (PwDs) to public universities on affirmative action.

This section describes experiences related to admission of students with disabilities to higher education. Admission in this paper refers to acceptance to enroll on a programme of study either through government sponsorship or on private sponsorship. Although education is guaranteed as a right (GoU, 1995), it is differentiated in this study for the purpose of who meets the costs of the study.

Admitted students are expected to receive a range of support services and systems to facilitate their academic and social inclusion. The outcome of this study indicated however that more students with disabilities experienced limited access to the academic programme of their first choice, office premises of the staff who should attend to their needs, information, auxiliary aids or systems and support services. It also appears that private universities and other government higher education institutions such as Uganda Colleges of Commerce (UCCs) and National Teachers Colleges (NTCs) rarely admit students with disabilities and other special needs through affirmative action. When these institutions have prior knowledge that the candidate has a disability the candidate is also not admitted. These limitations in the provisions are an obvious lack of reasonable accommodation necessary to enable students with disabilities and other special needs to manage learning and participation in different activities during their time in higher education.

Emong’s study reveals that, generally, a private university would only admit a student with disability on condition that it is able to meet the requirements of such a student. As noted by one private university official:

‘With the current facilities the university has and the existing staff knowledge on disabilities, we would not admit a blind or a deaf student. If they apply, they would be advised to join a university that has facilities catering for their needs.’

The argument for a university not to accept a student with disability when it does not have the required facilities would appear logically acceptable and realistic. Whereas the actions by the universities to fail or refuse to admit qualified candidates with disability amounts to direct discrimination of students with disabilities and contravenes section 6(2a) of the *Uganda Persons with Disabilities Act* (2006) and the spirit within the international human rights law. These scenarios point out that institutions of higher learning have inaccessible educational infrastructure, have not thought of support systems and services to enable students with disabilities to access learning like other students if admitted into the university and are not aware of the implications of the lack disability inclusion.

Findings also indicate that universities rarely consider admitting students with disabilities to specific programmes of their choices, especially purely science based or medicine disciplines. As noted by one of the officials responsible for admissions:

‘A person should be capable of physically doing a practical. One should be able to physically see and hear what is being examined during a practical. Thus, because of those conditions it is not advisable for a person with physical disability, visual impairment or hearing impairment to be enrolled for those courses as such a candidate cannot pass practical examinations.’

The opinion above depicts the understanding of disability by the official interviewed and its implications on the way disability inclusion in academic programmes in higher education is interpreted and can be attained. The opinion therefore merits analysis in relation to the meaning of disability. According to Barnes and Mercer (2010: 14-16) the treatment people with disabilities experience in society is informed by the meaning a given society or a service provider attaches to a disability. As Shakespeare (2006:272) points out, disability appears to refer ‘to limitation and incapacity, or to oppression and exclusion, or to both dimensions’.

If the opinion of the official above connotes disability to be a limitation or incapacity to perform - a view that implies that impairment and a disability are the same, a medical / individual model understanding of disability (Barnes and Mercer, 2010: 30-33) - then such a view potentially leads to exclusive discrimination of candidates with disabilities in some academic programmes. If the opinion considers disability as a result of barriers erected against students with impairments - a view that implies impairment and disability are distinct, a social model understanding of disability (Oliver, 2009) - then it calls for provisions of measures to eliminate barriers to participation by students with disabilities who qualify to join higher education. These measures are widely known as the provision of reasonable accommodation to people with disabilities (Lawson, 2008; Waddington, 2007) and are legally mandatory in accordance with the UN Convention on the Rights of Persons with Disabilities (CRPD) art. 24(5).

Whereas the provision of reasonable accommodation to people with disabilities is seen as a plausible solution to their exclusion in society, there are situations whereby their exclusion is largely arising from the intrinsic limitations associated with impairment. In other words, there are impairments in relation to some academic courses where no amount of environment change would eliminate a disadvantage associated with the impairment. This kind of scenario raises the case of disability inclusion in higher education beyond the arguments of the social model of disability to concur with its critics who argue that, in some instances, impairment can be real in the exclusion of people with disability (Crow, 1996; Abberley, 1987; Terzi, 2004; Bury, 2000; Thomas, 2002). It is important to note therefore, that, while the opinion of the academic participant quoted above might be true of particular academic programmes, interpreting it as broadly as it is presented may cause institutions of higher learning generally to discriminate against people with disability in some programmes, mainly science related and medical disciplines. Thus, the consideration of qualified people with disability to enroll in some academic programmes should be under case to case basis, taking into account how provision of reasonable accommodation as appropriate individualised support measures can facilitate their learning.

#### Support Services for students with disabilities

The study outcomes show that only public universities are providing support services to students with disabilities. However, the support services are only being provided to those students funded by government. While Naami (2015) found that PwDs who work experience problems at work, irrespective of their sex, disability type and employment sector, these challenges at work place can be equated to the experiences of lack of support needed at a study environment. Some of the support provided to students with disability is mainly personal assistance related support such as sighted guides for the blind, sign language interpreters for the deaf, helpers for those with physical disabilities and funds to purchase disability related devices, such as wheelchairs and Braille material. The mode of providing these supports is reported to differ in the public institutions. The variance could arise from the lack of institutional disability policy, the silence in the *Persons with Disability Act* (2006), lack of guidelines by the National Council for Higher Education on the required support students with disabilities and special needs in higher education should benefit from and lack of supervision on the support provided by each institution. As stated by one institutions of higher learning:

‘This university has no written policy on provision of support to students with disabilities. The current practice is based on the minutes of the University Council Meetings on the welfare of students with disabilities adopted at least 8 years ago.’

Arguably, if the said Acts had these provisions, then both public and private institutions of higher learning would be compelled to comply with the requirements. Without a policy on supporting students with disabilities, the support provided would be at the discretion of the officers of the institutions or any existing understanding for the support to these students. The implication is that the support is largely dependent on the good will of staff rather than on institutional policy. Literature available indicates that prevailing practices regarding disability in institutions of higher education are entrenched in medical rather than social frameworks (Collins & O’Mahony, 2001, Borland & James, 1999, Riddell, 1998, Reindal, 1995). Such individualised perspective and tendency makes support services and systems limited, making staff favour one impairment at the expense of the others, students fearing to be singled out and discriminated against or for students to negotiate their needs and services with individual staff. The implication is making the extent to which this support is provided inadequate. As expressed by one leader of students with disabilities:

‘The monetary value of the basic requirements for a blind student to effectively study exceeds far much the financial support he/she receives from the university. A blind student receives during the first year of his/her studies, 1,400,000/= Uganda Shillings (UGX). He/she is expected to buy; a Perkins machine which is 2,000,000/= UGX, a carton of Braille paper at 94,000/= UGX, Jaws computer software which is 2,300,000/= UGX, and a laptop computer which is at least 1,200,000/= UGX. For the student of limited mobility using a wheel chair, the cost of a new wheelchair is 400,000/= UGX and the university provides him/her 200,000/= UGX.’

Privately sponsored students, in particular those with hearing impairment, are unable financially to employ a sign language interpreter. As such, they either share such services with a student funded by government or study without. As noted by one student with hearing impairment:

‘Am told that the institution does not admit deaf people and that there are not provisions for interpreters. The lecturers claim they have no idea of how to help deaf students. We also miss out on group discussions. Lecturers for ICT are not considerate to us as explanations are made from any corner of the room and yet ICT is critical for our learning. I am likely to take longer on my Masters programme because of lack of accommodation.’

Data indicate that in one university, the plight of students who are deaf going without an interpreter attracted the intervention of one lecturer. The lecturer threatened to take legal action against the university over what he termed as ‘a gross violation of the rights of students with disabilities to education’. The lecturer noted:

‘It came to my attention that a privately sponsored student was attending lectures without the services of sign language interpreter. The university does not see it as its obligation to provide disability related support services to privately sponsored students. But the student had no money to employ the sign language interpreter and attending lectures without the sign interpreter was a disadvantage to him. I felt bad about this situation so I informed the university that I will secede from the university and take the university to court over violation of rights of the deaf students. That is when the university employed a sign language interpreter to the deaf student.’

Considering the cost of paying for an interpreter in addition to the tuition and recognising that an interpreter is the ear of the deaf student, the cost should be borne by the university in line with the requirement for reasonable accommodation. It is possible that students with deafness may be coming from poor families who can only pay for tuition, food and accommodation as a private student. This assumption is consistent with literature indicating that PwDs are more likely to be poor, especially in developing countries (WHO, 2011, Mitra *et al*, 2011, Kassah, 2008). It is important to note that 19.7% of Ugandans are poor and 42% of households earn their living from subsistence farming (UBOS, 2014). In addition, opinions on attitudes of the society towards people with disabilities was still negative (Masasa *et a*l, 2005). Thus, this study suggest that institutional policy needs to take into consideration provisions that are accommodative to private students taking cognisance of the fees that they pay, their family background and the right to education as enshrined in the international and national legislative documents.

### Physical accessibility

Access to physical facilities describes how students were reaching to and benefitting from library facilities and services, lectures, mode of delivery and mode of assessment. Generally, data indicated that physical accessibility was an overall impediment in all institutions of higher learning. Responses from all participants indicated that other than the recently constructed buildings, all the old buildings which are the bulk of lecture rooms in institutions of higher learning are to some extent inaccessible to people with mobility difficulties and other disabilities. They agreed that libraries have limited books and other publications. Because of the limited materials, restrictions are imposed on borrowing some of the library materials. There was acknowledgement of underdeveloped technological infrastructure, including internet services, in these institutions, which makes access to online materials very much limited. Participants argue that access to online academic resources in all universities is still being developed.

Data indicate that all students with disabilities find the libraries inaccessible in one way or another. For example, students with visual impairments find the materials in the libraries uniquely inaccessible. First, the restrictions on borrowing books pose greater challenges to blind students than other students, especially for books which are on the reserve selves. A blind student referring to this book has to Braille the material within the library, which is also an inconvenience to other library users arising from the condition that there must be silence in the library and the noise the brailing machine makes. A student with visual impairment recounts an experience:

‘I am not allowed to borrow a book and told to read in the library. I am not allowed to go in with my guide because she is not a student. In case I am allowed in with a guide, I am told the library is a quiet place and being read to by the guide is making noise to other readers. There is completely not consideration to my needs and yet am expected to perform at the same pace to other students.’

Second, the modifications being undertaken in some libraries such as the provision of ramps target mainly people with physical disabilities. No consideration is made to other impairments save to a limited extent, the Faculty of Special Needs and Rehabilitation library of Kyambogo University. Modifications could be done if students with disabilities were involved in decision making and proposing how such modifications can be made. The lack of involvement and accommodation in decision making is consistent with Naami’s (2015) study and World Health Organisation Report on Disability (2011). Thirdly, neither the institutions’ main libraries nor the departmental libraries have accessible publications such as brailed books and periodicals, audio recorded publications in tapes, CDs or accessible online journals. Data indicate that institutions recognise their obligation to provide equal library access to all library users and blame the failure of provision on lack of resources. Although, though this claim could be true it is possible to argue that the institutions may be lacking priority to disability inclusion.

#### Access to lectures, mode of delivery and assessment

Most storied buildings have never been modified. As experienced by a student with physical disability:

‘Fellow students are more aware of our disabilities and are prepared to help than the lecturers. A lecturer finds you struggling to climb the stairs and just passes by you and does not even show concern. When lecturer reaches the lecture room, he/she begins lecturing without bothering to wait for you to reach.’

Another student with physical disability states:

‘This semester I have missed 4 lectures because each time I went late, I felt it embarrassing and inconveniencing calling down my colleagues to carry me up. A class coordinator raised my concern to the head of the department during the first semester but to this end of the year, no response has been received.’

Section 26 of the *Persons with Disabilities Act* (2006) places a duty on the provider of a facility to make adjustments or to provide an alternative method of making the facility available to PwDs in cases where a physical feature such as one arising from the design or construction of a building or access to premises makes it impossible for PwDs to use that facility. While this does not require a provider to do anything which would fundamentally alter the nature of the service provided, the trade, profession or business, it is the inaccessibility that creates exclusion.

On learning and in assessment, data indicate that most institutions lack the facilities to support students with disabilities and other special needs. For example, lecture handouts which are mostly preferred by students with hearing impairment and students with physical disabilities were not easily available. For those lecturers who provide no Braille copies or put other accessible format were provided. Students with visual impairments feel lecture handouts can be a double cost in terms of time and money. Students with visual impairment have to Braille the handouts by themselves. Brailing requires a proficient reader which, most often, their guides are not, and as a result students with visual impairments rely on other students to read for them the print notes as they braille. An experience of a visually impaired student shows that:

‘One lecturer gave out notes for his module covering the whole semester, which was 300 pages. To transcribe that hand-out into Braille; means producing almost 1000 Braille papers of the notes. This requires a lot of time to do it and over relying on other students.’

While hearing students can get information informally from friends, students with hearing impairment need an interpreter or visual / print notices. Late posting and inaccessible notices limit information access of particularly deaf students. While dictation of notes is more favoured by the students with visual impairments, it is a great impediment for students with hearing impairment. The challenge arises when the lecturer talks and writes on the board at the same time. In this a way, a deaf student would have to balance between looking at the interpreter and the written work. There is often a lag in time which is not considered. To those who use a hearing aid, it may not be beneficial either. From the experiences of one student with hearing impairment:

‘The hearing aid is useless. It captures every sound in the hall. I have failed to determine a suitable position for myself to sit in the lecture halls, in order to hear lectures properly. Every side I try I cannot properly hear the lectures. The worst part is, even the height of a lecturer sometimes makes it difficult for me to hear the lecture. More so I do not even copy notes as most of the time lecturers dictate notes. I rely on photocopying notes from other students. In that respect, I spend a lot of money in photocopying.’

Another student with hearing impairment stated:

‘I feel the lecturers have not understood our constraints. For me I don’t get information through dictation but that is the order of the day and that is what the lecturers are used to. In one of the assessment tests, the lecturer made corrections verbally as such I did not get the corrections- he informed the students that in number…, a zero is missing so please add it in front of that figure…. The other was when another lecturer gave us course work of 2 numbers. The verbal instructions were, “one number was to be done there and then as test; the other number was a take home course work.” Because it was verbal instructions I did not hear it, as a result I did both numbers as a test. In some lectures, when I beg for pardon, the response is “I do not repeat.”’

Section 21 of the PwDA lays a duty on the responsible government authority to promote the rights of persons with disabilities to access information through the development and use of sign language, tactile and sign language interpreters, in all public institutions and at public functions, and brailling of public information, such as government documents, government newspapers and other publications. Although the Act is silent on providing relevant information like government documents electronically to those who cannot read Braille, the demand is an international obligation that Uganda is a signatory to.

A similar challenge extends to examination as most examinations are mostly in print and institutions find it challenging transcribing brailed work into print for marking. The setting of questions takes limited consideration for the varying special needs of students.

A visually impaired student recounts:

‘In one semester, I was forced to do only questions in section 1 as most questions in section 2 were mainly practical. I felt the examinations were hard for me. I felt again that my former secondary school is better than this university in understanding my disability as it was brailing examinations for me. This university finds it challenging to transcribe brailed works into print as a result; blind students do exams for the second semester when they have not known the results for the first semester examination including course work results. Examinations are not brailed. I feel it is unethical. During examination we are asked to Braille paper before doing it.’

Section 20 of the PwDA requires all public buildings to be accessible to all sections of the public who are invited to it and places a duty on the owners of public buildings to ensure this. The public buildings should have an accessible entrance, accessible pathways and accessible elevators. They should also have accessible toilets for diverse disabilities, and well-dimensioned staircases and ramps for people with mobility difficulty or in wheelchairs. The PwDA requires that adequate railing should be provided around stairs, ramps and raised platforms. Multi-storied buildings must have well-dimensioned elevators for convenient use by people with disabilities. The elevators should have embossed numerals on selector buttons and arrival signals to cater for visually impaired and deaf passengers simultaneously. The law also demands that ‘where it is difficult or unfeasible to install a ramp or an elevator to an existing building the owner of building shall provide platform lifts to provide accessibility’.

Although the experiences of students with disabilities in higher education depict generally their exclusion in the institutions, there are positive efforts to help the situation, which need to be enhanced. First, there is willingness among some staff to promote disability inclusion. Second, there are some internal initiatives to support students with disabilities at faculty level in some universities. However, such support is mainly reactive ‘reasonable accommodation’ and appears to be dependent on the good will of the individual lecturers and not structurally framed within a formal policy. The experience at faculty level is that each faculty determines what to do when confronted with the needs of a student with disability; a practical faculty acknowledged a challenge to implementation, as seen from this excerpt:

Nothing special offered to students with disabilities, except we are considerate when setting level of achievement for practical activities. Students with disabilities though not officially given concessions during practical classes and during assessment of practical are considered differently depending on their disability.

Section 27 of the Act specifies that it shall be the duty of the providers of services to provide auxiliary aid or service where it enables or facilitates PwDs to make use of a service. Such services include sign language interpreters, sighted guides, wheelchair guides, readers and transcribers.

It is arguable that lack of proactive planning for students with disabilities is attributed to the overall limited resources within which institutions of higher learning operate.

We shall deal with barriers affecting students with disabilities as we receive students with disabilities. It is difficult to anticipate the barrier and plan for its removal within the limited resource environment we operate.

Overall, lack of equal opportunities on PwDs and limited knowledge about disability in higher education has disadvantaging effects to some students with disabilities in some programmes. In some cases, some are forced to terminate their studies. Kwesiga and Ahikire (2006), cite a student whose studies were terminated due to prejudices about the cause of disability stating:

There was also a case of a lame student who had to drop out of medicine at the third year because the instructors demanded so. According to one deputy registrar the student progressed well until she reached the stage for clinicals, and the lectures were of the view that clinicals and crutches could not go together.

## Conclusions and recommendations

The findings in this article, although they cannot be generalised, are expected to contribute to the theoretical discourse on disability inclusion in higher education in Uganda. The experiences and voices presented herein have implications on informing institutional policies and practices, not only in Uganda but regionally, in relation to including students with disabilities in higher education. Disability inclusion in higher education will contribute to achieving the Sustainable Development Goals, particularly Goals 1, 3, 4, 5 and 8, and other international and national policy provisions aimed at contributing towards poverty alleviation that the government of Uganda advances.

Cognisant that institutions are providing some support to students with disabilities, it can be argued in this article that there is some level of awareness by institutions of higher learning in Uganda on the matter of disability inclusion. This awareness has however not been enhanced, probably due to the lack of established mechanisms for mainstreaming disability in higher education. The lack of established mechanisms can be attributed to the lack of guidelines or directives on disability inclusion for higher education. As such, there is need for policy directives requiring institutions to adopt reasonable accommodation and other equality of opportunities measures for students with disabilities.

The Uganda National Council of Higher Education and the Ministry of Education, Science, Technology and Sports are mandated to come up with guidelines on support services that institutions of higher education should provide to students with disabilities and other special educational needs. These guidelines should arise from the national and institutional policy provisions on disability inclusion in higher education. Institutions of higher learning, as a matter of quality assurance, should be required to develop a Institutional Disability Policy and strategic plans to implement it. Government should include in her funding provisions a vote on disability inclusion. Disability awareness should be a strategy that is created across all units, including among students, staff and visitors to the institutions.

Effecting disability inclusion will require that higher education institutions are compelled to collect data on students with disability and other special educational needs, and document their experiences to facilitate planning. Collaboration with DPOs and the Equal Opportunities Commission on matters of data collection and creating disability awareness in institutions of higher learning is a necessity. The involvement of DPOs is in line with the slogan ‘*Nothing for us without us’* and the stronger principle of the proposed Sustainable Development Goals -‘*leave no one behind’*, the expertise and experience they have and the requirements of the CRPD. The Equal Opportunities Commission (EOC) is mandated to bring about equal opportunities in all institutions and organisations.

As a sustainable strategy for disability inclusion in higher education, universities and other institutions of higher learning should establish a Disability Support Centre. A Disability Support Centre is a critical and an important infrastructure of the institutions in bringing about disability equality in the institution. The Disability Support Centre will be a disability think tank for the institutions regarding disability inclusion and advising on disability mainstreaming. The support center also becomes a focal point for collaboration with stakeholders; a place for assessment of disability and providing advice to respective units within the University accordingly. The center should therefore be managed by staff with the requisite professional background, knowledge, skills and attitude.

## Footnotes

1 An earlier version of this article was presented to the Ministry of Education and Sports and the National Council for Higher Education, Uganda.

2 See the wording of section 28 of the Universities and other Tertiary Institutions Act 2001 (as amended).

3 See UN Committee on Economic, Social and Cultural Rights (CESCR), General Comment No. 11: Plans of Action for Primary Education (Art. 14 of the Covenant), 10 May 1999, E/1992/23, available at: http://www.refworld.org/docid/4538838c0.html [accessed 1 May 2016].

4 See the Universal Declaration of Human Rights (UDHR), Art 26(1).

5 UN Committee on Economic, Social and Cultural Rights (CESCR), General Comment No. 13: The Right to Education (Art. 13 of the Covenant), 8 December 1999, E/C.12/1999/10, para. 6.

6 See article 13 of the International Covenant on Economic, Social and Cultural Rights (ICESCR).

7 See the UN Charter Art 1(3) and 55 (3) and UN Bill of Human Rights i.e. the Universal Declaration of Human Rights (UDHR) Art 2, the International Covenant on Civil and Political Rights (ICCPR) Art 2(1) and the International Covenant on Economic, Social and Cultural Rights (ICESCR) Art 2(2).

8 See also the preamble of the Universal Declaration of Human Rights (UDHR) Art 1.

9 Other legal instruments of the draft Special Needs and Inclusive Education policy (2011).

10 Universities and Other Institutions Act (2011) S. 5(g);

11 S. 5(l);

12 S. 112(2)(e) and (i);

13 S. 117/

14 S. 38(1)(r);

15 UTIA s 24(1) (b).

16 Ibid s 24(1) (c).

17 Ibid s 5.

18 Ibid s 6.

19 Ibid Part iv.

20 Ibid, second Schedule parts 7 and 8.

21 Ibid S.6.

22 Prior to the structural adjustments to economic policies, there was only one state university, Makerere University, and 33 other tertiary institutions of education. see the National Council for Higher Education, ‘Recognised Universities 2010’. http://www.unche.or.ug/page2.php?nid=27

23 The author estimates this number from table 2.2.8: Secondary School Students with Special Needs, (2007-2010) of the Uganda Statistical Abstract 2013. It is indicated that in 2010, there were 843 students with special needs in S.6.
